# Global, regional, and national burden of orofacial clefts, 1990–2021: an analysis of data from the global burden of disease study 2021

**DOI:** 10.3389/fmed.2025.1609700

**Published:** 2025-06-11

**Authors:** Zhenghao Wang, Weikun Qi, Yiru Chen, Feng Niu

**Affiliations:** Plastic Surgery Hospital, Chinese Academy of Medical Sciences and Peking Union Medical College, Beijing, China

**Keywords:** cleft lip and palate, GBD (2021) database, disease burden analysis, prevalence, incidence, DALY (disability-adjusted life years)

## Abstract

**Background:**

Orofacial clefts (OFCs) are congenital craniofacial malformation caused by embryonic developmental abnormalities, characterized by incomplete fusion of the upper lip and/or palate, leading to feeding difficulties, speech impairments, and other functional challenges. OFCs represent the most prevalent congenital malformations of oral and maxillofacial region. We aim to characterize disease burden of OFCs across regions and countries, analyze temporal trends from 1990 to 2021, examine relationship with Socio-demographic Index (SDI), explore gender disparities and predict future epidemiological patterns.

**Methods:**

Utilizing GBD 2021 database for 204 countries/regions, we analyzed age-standardized metrics including disability-adjusted life years (DALYs), prevalence, mortality, incidence, using tools like DisMod-MR 2.1 for Bayesian meta-regression. SDI, calculated from educational attainment, per capita income, and fertility rates (range 0–1), stratified nations into quintiles. Statistical analyses included SDI-burden correlations and future projections using Bayesian age-period-cohort (BAPC) modeling, implemented through R software.

**Results:**

In 2021, there are a total of 4124006.8 cases of OFCs worldwide, with an age-standardized prevalence rate (ASPR) of 53.4 per 100,000 (95% UI: 43–64). The age-standardized incidence rate (ASIR) was 3.0 per 100,000 (95% UI: 2.2–3.9), while age-standardized deaths rate (ASDR) of 0 per 100,000 (95% UI: 0–0.1). Additionally, age-standardized DALYs rate was 5.8 per 100,000 (95% UI: 3.5–9.8). Regionally, low- to middle-SDI regions demonstrated the highest ASPR and ASIR, whereas low-SDI areas showed the most severe ASDR and DALYs rate. In contrast, high-SDI regions consistently exhibited the lowest burden across all metrics. At the subregional level, South Asia recorded the greatest ASPR, while Central Asia had the peak ASIR. Oceania displayed the highest ASDR and DALYs rate. Country-specific analysis identified Palestine with the maximum ASPR, Kazakhstan with the highest ASIR, Papua New Guinea with the greatest ASDR, and Afghanistan with the most elevated DALYs rate.

**Conclusion:**

The global OFCs burden demonstrated consistent decline from 1990–2021, with persistent male predominance. Regional disparities correlate strongly with SDI, particularly affecting Central Asia, South Asia, and Africa populations.

## Introduction

Orofacial clefts (OFCs) is the most prevalent congenital maxillofacial malformation (1), which with both genetic and environmental etiological factors implicated in their pathogenesis. The embryological basis of OFCs formation occurs during critical facial morphogenesis (weeks 4–12 of gestation) (2), when pathogenic genetic variations in multiple genes disrupt essential signaling pathways such as BMP, TGF-*β*, and WNT, affecting facial development during this stage and causing the occurrence of OFCs. In addition, advanced maternal age or early pregnancy with viral infections, consanguinity, ionizing radiation, and environmental pollutants, deficiencies in folate and vitamins, use of antiepileptic drugs and steroids, maternal metabolic disorders, endocrine abnormalities, parental occupational pesticide exposure, lifestyle factors can increase the likelihood of OFCs (3–12).

OFCs are clinically categorized into two primary subtypes: syndromic forms (co-occurring with additional congenital defects such as cardiac or limb abnormalities) and non-syndromic isolated cases. The treatment of OFCs involves primary lip/palate repair, secondary alveolar bone grafting, orthodontic intervention, orthognathic surgery, respiratory and pronunciation training, otolaryngology treatment, which requires collaboration among disciplinary teams (such as oral and maxillofacial surgery, orthodontics, speech therapists, etc.) (13) and treatment protocols must be carefully staged according to the patient’s developmental stage. A complete treatment requires substantial financial resources and prolonged treatment duration (14).

At the physiological level, patients with OFCs frequently experience oral dysfunction, including impaired sucking ability leading to feeding difficulties, cleft palate leading to coughing and otitis media risks, speech impairments affecting approximately 70% of cases. Psychologically, patients are prone to developing feelings of inferiority due to differences in appearance, while long-term psychological stress may predispose patients to anxiety, depression and other mood disorders. Research further indicates that patients with OFCs have lower average academic achievement, particularly those with cleft palate (15–17). Additionally, the condition imposes socioeconomic constraints by limiting career options (e.g., broadcasting, flight attendant, etc.) and barriers to professional advancement (18).

Previous studies have found that the disease burden of OFCs is related to subject characteristics, such as gender, region, social development level (2, 19), but there is a lack of comprehensive and specific description and analysis. This study analyzes data from GBD database with the aim of obtaining a detailed, quantitative, comprehensive understanding of the disease burden of OFCs, and making predictions on its development trend at the global level. We hope to provide some reference for reducing the disease burden in different regions.

## Method

### Data sources

Data is sourced from GBD database (2021), which is a global health research platform supported by Institute for Health Metrics and Evaluation (IHME) at the University of Washington, and jointly maintained by World Health Organization (WHO), the World Bank, and other institutions. It covers the health data of 204 nations and territories worldwide since 1990, quantifies 87 risk factors and 371 diseases, meanwhile providing standardized indicators such as age standardized rate (ASR) multi-dimensionally classified such as age, gender, and year. The GBD 2021 study applies complex statistical models such as MR-BRT, DisMod MR 2.1, CODEm, etc. to adjust the collected data from around the world to reduce its heterogeneity. We obtained processed data (prevalence, incidence, deaths, and DALYs) for all regions and countries from the GBD 2021 study for secondary analysis.

### SDI

SDI serves as a composite metric for assessing regional socioeconomic development status. This index, ranging from 0 (minimal development) to 1 (optimal development), is derived from three weighted components: gross domestic product per capita, educational attainment (measured by average schooling years), and fertility rates. Based on their SDI scores, geographical units were stratified into five distinct development tiers (high, high-middle, middle, low-middle, low). The focus of this study is on relation between SDI values and various disease burden indicators.

### Statistical analyses

Disease burden metrics selected for this study comprise four principal indicators: “Prevalence,” “Incidence,” “Deaths” and “DALYs.” ASR adjusts crude rate (including incidence rate and mortality) by applying a standardized population distribution. This methodological approach effectively eliminates potential biases arising from differences in population age distributions between regions or across time periods. The ASR per 100,000 individual is computed according to equation below: $\text{ASR}=\frac{\sum_{i=1}^{A}a_{i}w_{i}}{\sum_{i=1}^{A}w_{i}}$×100,000 (a_i_: the i^th^ age category’s rate in specific age range; w: corresponding i^th^ age group’s population size within reference population; A: total count of group). From GBD database, age-standardized death rate (ASDR), age-standardized prevalence rate (ASPR), age-standardized DALYs rate, and age-standardized incidence rate (ASIR).

The temporal trends of ASR were quantified with estimated annual percentage change (EAPC). The computational approach involves fitting the model: ln(y_t_) = *α* + *β*t + *ϵ* (y_t_ is the natural logarithm of ASR; α is the intercept; β captures the temporal slope coefficient; ϵ accounts for random variation). The EAPC value is subsequently calculated as 100 × [exp(*β*)−1]. Linear regression model is applied for calculation on 95% confidence interval (CI) of EAPC. If the lower bounds of EAPC with its entire 95%CI are both positive, it indicates a significant upward trend in ASR. On the contrary, if the upper bounds of EAPC and its 95%CI are both negative, it reflects a significant downward trend in ASR. If both conditions are not met, the ASR is defined as stable.

We conducted correlation analysis on the disease burden (ASPR, ASIR, age-standardized DALYs rate, ASDR) and SDI values of all regions and countries, which were performed in R version 4.4.2. We use “cor.test” function from “stats” R package to carry out Pearson correlation analysis. All tests were two-sided with a significance level set at *p* < 0.05. In addition, we conducted statistical analysis on the disease burden of different genders worldwide using “tbl_summary” function from “gtsummary” R package.

### BAPC

The Bayesian age–period–cohort (BAPC) model is an analysis tool based on the Bayesian statistical framework, mainly used to decompose the age, period, and queue effects of disease burden over time, and predict future trends. The statistical principle is based on Bayesian inference, using Markov Chain Monte Carlo (MCMC) or Integrated Nested Laplace Approximation (INLA) algorithms to handle data loss and uncertainty, while introducing prior knowledge to enhance the robustness of small sample data. Compared to traditional models, its advantages lie in the comprehensive analytical ability of multidimensional time effects, robust handling of data sparsity and measurement errors, and flexible integration of multidimensional data. In this study, we applied disease burden data from GBD 2021 and demographic forecast data from the IHME, using the “BAPC” R package to predict the trend of disease burden changes at the global level.

## Result

### Global level

**Figure 1:**
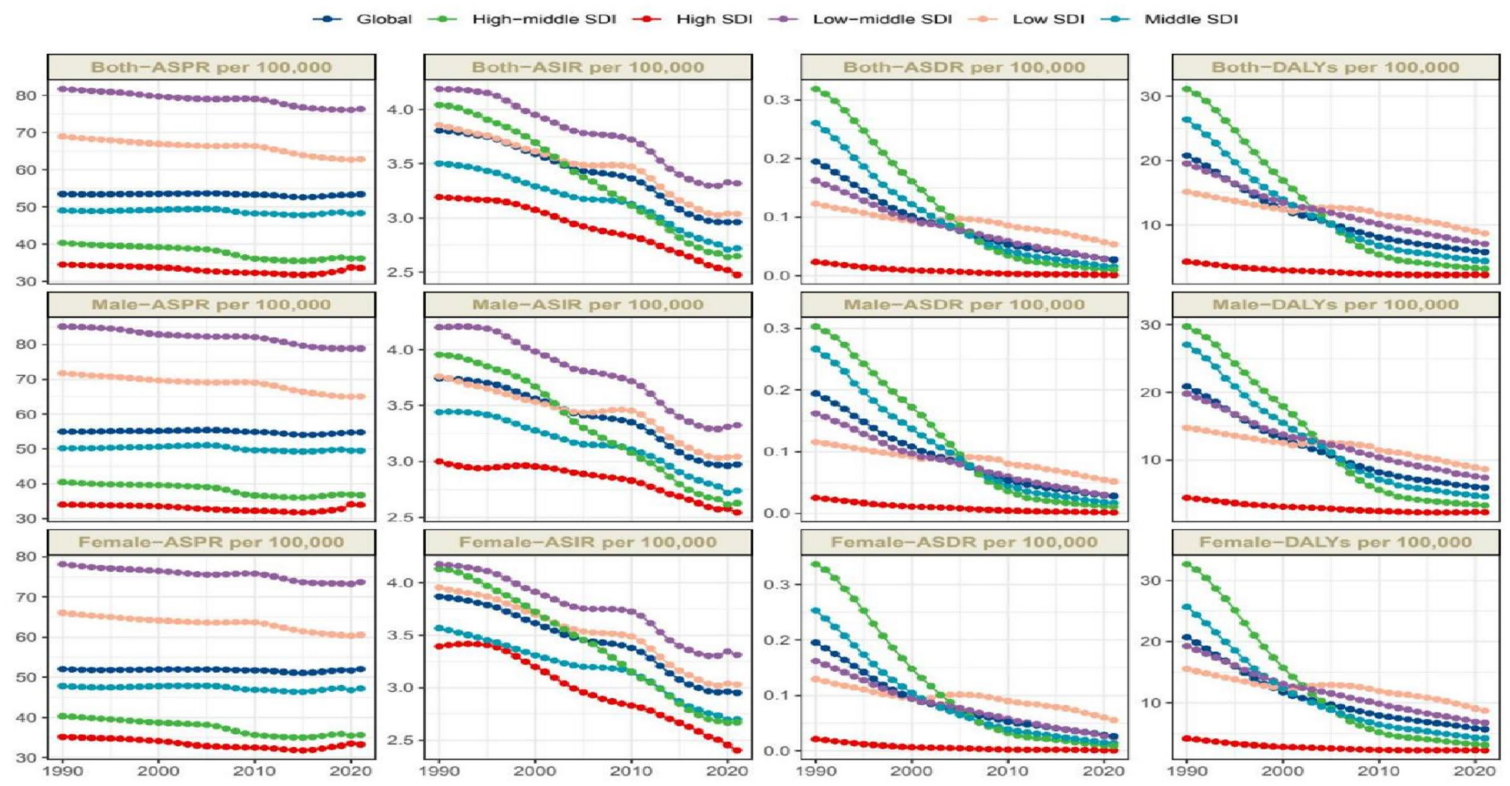
Trends in OFCs disability-adjusted life-years, OFCs prevalence, deaths, and incidence (1990–2021).

Global prevalence of OFCs reached 4,124,006.8 cases (95%UI: 3,318,692.5–5,026,199.6) in 2021, representing a 40.3% increase from 1990 (2,937,706.5 cases, 95% UI: 2,389,357.8–3,535,593.7). Despite this substantial growth in case number, ASPR in 2021 remained nearly unchanged at 53.4 per 100,000 (95% UI: 43–65), comparable to the 1990 ASPR of 53.5 (95% UI: 43.4–64.5). EAPC of ASPR was −0.04 (95% CI: −0.05 to −0.02) (Table 1; Figure 1). Global incidence of OFCs in 2021 was 183,302.4 cases (95%UI: 135,255.4–241,690.8), marking a 24.79% decrease from 1990. ASIR declined from 3.8 per 100,000 (95% UI: 2.8–4.8) in 1990 to 3.0 per 100,000 (95% UI: 2.2–3.9) in 2021. EAPC for ASIR was −0.89 (95% CI: −0.94 to −0.83), suggesting a persisting downward trend in incidence (Table 1; Figure 1). OFCs-related deaths were estimated at 1,718.6 cases (95% UI: 484.8–4,409.5) in 2021, reflecting an 86.09% reduction compared to 1990, with an EAPC of −6.17 (95% CI: −6.24 to −6.11) (Table 1; Figure 1). Global DALYs attributed to OFCs reached 408,775.3 (95% UI: 252,320.1–671,119.9) in 2021, showing a 68.32% decrease from 1990. The age-standardized DALYs rate stood at 5.8 per 100,000 (95% UI: 3.5–9.8), with an EAPC of −4.13 (95% CI: −4.28 to −3.98) (Table 1; Figure 1).

**Table 1:** Trends in global and regional impact of OFCs: disability-adjusted life years, mortality, incidence, and prevalence (1990–2021).

Location	1990		2021		EAPC_95%CI
	Number	ASR	Number	ASR	
Prevalence					
Global	2937706.5 (2389357.8–3535593.7)	53.5 (43.4–64.5)	4124006.8 (3318692.5–5026199.6)	53.4 (43–65)	−0.04 (−0.05 to −0.02)
High SDI	290059.3 (236919.9–349,088)	34.5 (28.3–41.3)	343810.2 (275876.4–419985.2)	33.6 (27–40.6)	−0.23 (−0.29 to −0.16)
High-middle SDI	421007.6 (343062.6–503601.7)	40.4 (32.9–48.3)	436311.1 (356149.1–530335.5)	36.2 (29.6–43.5)	−0.46 (−0.52 to −0.39)
Middle SDI	865024.7 (699926–1041409.4)	49 (39.6–59.1)	1148111.4 (927870.2–1,400,797)	48.4 (39.2–58.8)	−0.07 (−0.1 to −0.04)
Low-middle SDI	991,187 (790214.7–1209512.2)	81.7 (64.6–99.9)	1470278.2 (1169396.5–1805705.6)	76.3 (60.7–93.6)	−0.23 (−0.25 to −0.22)
Low SDI	368724.4 (296834.5–446,755)	68.9 (55–83.8)	722,909 (583611.3–879494.8)	62.8 (50.5–76.8)	−0.29 (−0.32 to −0.26)
Andean Latin America	17211.4 (13884.4–20618.5)	42.7 (34.3–51.6)	33534.4 (27121.2–40,759)	51.1 (41.4–62)	0.6 (0.54 to 0.65)
Australasia	6162.6 (5215.5–7250.8)	31.6 (26.8–37)	8317.2 (6674.3–10154.6)	28.6 (22.9–34.6)	−0.28 (−0.31 to −0.24)
Caribbean	11081.1 (8958.3–13239.6)	30.7 (24.7–36.9)	21561.2 (17052.5–26,692)	46.2 (36.6–57)	1.63 (1.54 to 1.72)
Central Asia	66348.4 (53898.9–78498.1)	91.7 (74.3–109.5)	76835.2 (62046.7–92647.7)	80 (64.6–96.4)	−0.45 (−0.51 to −0.39)
Central Europe	41791.8 (34048.2–49369.6)	34.9 (28.4–41)	30463.8 (24477.3–36896.1)	28.4 (23–34.2)	−0.62 (−0.66 to −0.58)
Central Latin America	80082.1 (65637–95633.8)	46 (37.8–55.5)	96,757 (78596.3–117245.7)	39.1 (31.9–47.3)	−0.52 (−0.55 to −0.48)
Central Sub-Saharan Africa	24064.8 (19126.8–29127.3)	39.4 (31–48)	55173.7 (44008.9–68507.1)	38.3 (30.6–47.8)	−0.03 (−0.06 to 0.01)
East Asia	462829.9 (373116.8–559914.1)	38.2 (30.8–46.2)	429540.6 (347839.7–519201.4)	31.1 (25.5–37.1)	−0.86 (−1.02 to −0.71)
Eastern Europe	69926.1 (56139.6–85059.8)	32 (25.7–38.8)	52,313 (41814.6–64112.7)	26.8 (21.5–32.6)	−0.57 (−0.58 to −0.55)
Eastern Sub-Saharan Africa	110129.2 (88036–132914.8)	52.5 (41.4–64)	221976.3 (179210.7–271859.5)	49.7 (39.8–61.3)	−0.18 (−0.23 to −0.14)
High-income Asia Pacific	91322.8 (74154.6–109,942)	55.9 (45.3–67)	90815.2 (71751.2–111736.4)	52.9 (42.3–64.6)	−0.22 (−0.24 to −0.2)
High-income North America	52229.1 (39822–66060.5)	19.2 (14.6–24.3)	68252.3 (52114.7–86705.8)	19.7 (15.1–24.9)	−0.5 (−0.81 to −0.19)
North Africa and Middle East	317508.8 (256926.6–380861.3)	89.4 (72–107.5)	532165.9 (425774.6–646873.5)	85.2 (68.2–103.5)	−0.11 (−0.15 to −0.08)
Oceania	1764.8 (1396.1–2158.5)	25.7 (20.3–31.6)	4786.2 (3804.6–5947.7)	33.4 (26.3–41.5)	0.82 (0.78 to 0.86)
South Asia	1134885.4 (893798–1388417.5)	100.2 (78.7–123.2)	1633290.6 (1295458.2–2022505.8)	89.1 (70.7–110.1)	−0.42 (−0.44 to −0.4)
Southeast Asia	137134.4 (110284.9–165525.5)	28.5 (23–34.6)	294296.5 (238649.7–359900.3)	43 (34.9–52.4)	1.32 (1.24 to 1.4)
Southern Latin America	13501.2 (10784–16292.9)	27.1 (21.7–32.7)	16307.1 (12730.3–19984.6)	25.4 (20–31)	−0.23 (−0.36 to −0.1)
Southern Sub-Saharan Africa	33516.9 (26947.1–40948.2)	61.1 (48.9–75)	46724.7 (37840.3–57921.6)	58.1 (47.1–71.8)	−0.15 (−0.18 to −0.12)
Tropical Latin America	45985.2 (37322.9–55399.5)	29.7 (24.1–35.8)	53196.6 (43518.5–63293.2)	24.1 (19.8–28.7)	−0.64 (−0.82 to −0.47)
Western Europe	110914.7 (92933.7–130198.3)	30.8 (25.8–35.8)	106619.5 (86208.2–128179.7)	26.6 (21.6–31.6)	−0.61 (−0.68 to −0.55)
Western Sub-Saharan Africa	109315.9 (87287.1–132892.3)	51.6 (41.1–63.4)	251079.8 (202129.3–306333.6)	48.2 (38.5–59.2)	−0.16 (−0.2 to −0.12)
Incidence					
Global	243735.5 (179582.3–309986.2)	3.8 (2.8–4.8)	183302.4 (135255.4–241690.8)	3 (2.2–3.9)	−0.89 (−0.94 to −0.83)
High SDI	19184.2 (14943.5–23,686)	3.2 (2.5–3.9)	12250.7 (8958.3–15793.5)	2.5 (1.8–3.2)	−0.84 (−0.89 to −0.78)
High-middle SDI	35467.7 (27356.6–44190.3)	4 (3.1–5)	14891.1 (10856.4–19103.2)	2.6 (1.9–3.4)	−1.53 (−1.59 to −1.47)
Middle SDI	70,155 (52194.6–89621.5)	3.5 (2.6–4.5)	41628.6 (30595.4–54118.8)	2.7 (2–3.5)	−0.83 (−0.89 to −0.77)
Low-middle SDI	77840.5 (55680.3–103242.8)	4.2 (3–5.6)	61922.3 (45962.8–82936.3)	3.3 (2.5–4.4)	−0.86 (−0.92 to −0.81)
Low SDI	40949.9 (28498.5–53784.9)	3.9 (2.7–5.1)	52492.5 (37730.8–70330.4)	3 (2.2–4.1)	−0.8 (−0.86 to −0.73)
Andean Latin America	2389.1 (1779.9–3030.2)	4.2 (3.2–5.4)	2073 (1528.4–2708.1)	3.5 (2.6–4.6)	−0.86 (−0.95 to −0.77)
Australasia	523.9 (476.5–581.8)	3.4 (3.1–3.8)	446 (310.4–596.5)	2.6 (1.8–3.5)	−0.69 (−0.91 to −0.47)
Caribbean	930.2 (637.9–1,240)	2.2 (1.5–2.9)	836.3 (589.8–1178.5)	2.2 (1.5–3.1)	0.28 (0.16 to 0.41)
Central Asia	7838.8 (5676.3–9950.7)	8.3 (6–10.5)	5026.9 (3489.7–6802.2)	5.1 (3.6–6.9)	−1.75 (−1.83 to −1.67)
Central Europe	2950.7 (2260.7–3684.6)	3.6 (2.7–4.5)	1199.3 (846.9–1582.1)	2.4 (1.7–3.1)	−1.2 (−1.29 to −1.11)
Central Latin America	9706.6 (7405.6–12225.3)	4.1 (3.1–5.1)	4740.3 (3408.8–6221.5)	2.5 (1.8–3.3)	−1.25 (−1.35 to −1.14)
Central Sub-Saharan Africa	3733.3 (2516.1–4946.6)	3 (2–4)	4471.9 (3198.6–6112.9)	2.1 (1.5–2.9)	−1.27 (−1.33 to −1.21)
East Asia	43150.1 (32544.5–53678.5)	3.8 (2.8–4.7)	12,651 (9475.1–16014.8)	2.3 (1.7–2.9)	−1.72 (−1.92 to −1.52)
Eastern Europe	3774.3 (2719.4–4815.6)	2.6 (1.9–3.4)	1586.6 (1121.2–2126.9)	1.8 (1.3–2.5)	−1.19 (−1.26 to −1.13)
Eastern Sub-Saharan Africa	15197.8 (10377.7–20,427)	3.5 (2.4–4.7)	18546.2 (13042.2–25,247)	2.8 (2–3.8)	−0.66 (−0.8 to −0.52)
High-income Asia Pacific	5042.6 (3779.5–6350.1)	5.3 (4–6.7)	2180.6 (1421.8–2947.3)	3.8 (2.5–5.2)	−1.14 (−1.19 to −1.09)
High-income North America	3937.7 (2830.5–5155.4)	1.8 (1.3–2.3)	3367.6 (2357.8–4455.8)	1.7 (1.2–2.3)	0.01 (−0.09 to 0.12)
North Africa and Middle East	25886.2 (18883.2–33252.8)	4.9 (3.6–6.3)	18033.9 (13630.9–23855.2)	3.2 (2.4–4.2)	−1.44 (−1.52 to −1.37)
Oceania	138.4 (93.4–197.2)	1.3 (0.9–1.8)	299 (211.2–431.2)	1.5 (1–2.1)	0.38 (0.3 to 0.46)
South Asia	74558.2 (53276.1–102626.7)	4.5 (3.2–6.2)	57043.1 (41548.6–77685.2)	3.8 (2.7–5.1)	−0.77 (−0.82 to −0.72)
Southeast Asia	13065.7 (9327–17114.9)	2.2 (1.6–2.9)	14629.7 (10515.7–19032.5)	2.7 (1.9–3.5)	0.67 (0.62 to 0.73)
Southern Latin America	1444.9 (1080.2–1872.5)	2.8 (2.1–3.7)	764.4 (519.4–1013.9)	2.1 (1.4–2.7)	−1.08 (−1.47 to −0.69)
Southern Sub-Saharan Africa	2960.6 (2068.9–4,011)	3.8 (2.7–5.2)	2587.4 (1853.9–3470.3)	3.3 (2.4–4.4)	−0.41 (−0.47 to −0.35)
Tropical Latin America	3782.9 (2986.2–4683.2)	2.3 (1.9–2.9)	3344.2 (2530–4235.2)	2 (1.5–2.6)	−0.18 (−0.3 to −0.05)
Western Europe	7394.4 (6172.4–8535.2)	3.3 (2.8–3.8)	4963.5 (3699.8–6228.8)	2.5 (1.9–3.2)	−0.9 (−0.95 to −0.85)
Western Sub-Saharan Africa	15329.2 (10349.3–20589.2)	3.6 (2.4–4.8)	24511.6 (17346.4–33092.8)	2.9 (2–3.9)	−0.61 (−0.68 to −0.53)
Deaths					
Global	12353.8 (4489.9–22,917)	0.2 (0.1–0.4)	1718.6 (484.8–4409.5)	0 (0–0.1)	−6.17 (−6.24 to −6.11)
High SDI	140.1 (53.3–265.1)	0 (0–0)	6.6 (3.1–11.4)	0 (0–0)	−8.59 (−8.81 to −8.37)
High-middle SDI	2818.1 (1099.6–5607.2)	0.3 (0.1–0.6)	57.2 (30.9–90.8)	0 (0–0)	−11.62 (−12.12 to −11.11)
Middle SDI	5,193 (2311.3–8942.5)	0.3 (0.1–0.4)	238.3 (141.5–360.4)	0 (0–0)	−9.04 (−9.21 to −8.86)
Low-middle SDI	2952.8 (850.8–7563.3)	0.2 (0–0.4)	496.7 (176.9–1068.9)	0 (0–0.1)	−5.45 (−5.63 to −5.28)
Low SDI	1243.7 (102.7–4732.9)	0.1 (0–0.5)	917.2 (110.9–3315.9)	0.1 (0–0.2)	−2.1 (−2.36 to −1.85)
Andean Latin America	182.6 (87.2–307.8)	0.3 (0.2–0.6)	12.6 (7.1–20.1)	0 (0–0)	−8.73 (−9.03 to −8.43)
Australasia	0.3 (0.2–0.5)	0 (0–0)	0.2 (0–0.5)	0 (0–0)	−3.93 (−9.13 to 1.56)
Caribbean	20.5 (5.1–53)	0 (0–0.1)	8.1 (1.7–20.1)	0 (0–0.1)	−2.45 (−2.56 to −2.34)
Central Asia	27.2 (14.8–51)	0 (0–0.1)	9.6 (5.7–16.6)	0 (0–0)	−4.15 (−4.93 to −3.37)
Central Europe	19.1 (12.2–29.1)	0 (0–0)	0.3 (0.1–0.6)	0 (0–0)	−11.81 (−12.75 to −10.86)
Central Latin America	288.9 (171.3–369.1)	0.1 (0.1–0.2)	41.2 (24.2–60.2)	0 (0–0)	−5.67 (−5.87 to −5.47)
Central Sub-Saharan Africa	80.9 (8–357.2)	0.1 (0–0.3)	58.5 (7.2–239.7)	0 (0–0.1)	−2.33 (−2.9 to −1.76)
East Asia	5997.5 (1999–11610.8)	0.5 (0.2–1)	103 (52.1–169.7)	0 (0–0)	−11.52 (−12.21 to −10.81)
Eastern Europe	29.8 (18.4–43.2)	0 (0–0)	2.1 (0.8–3.5)	0 (0–0)	−7.48 (−8.21 to −6.75)
Eastern Sub-Saharan Africa	455 (44.4–2,137)	0.1 (0–0.5)	257 (26.7–1121.5)	0 (0–0.2)	−2.84 (−3.05 to −2.63)
High-income Asia Pacific	16.6 (6–36.6)	0 (0–0)	0.1 (0–0.1)	0 (0–0)	−16.54 (−17.79 to −15.26)
High-income North America	13.9 (10.3–16.4)	0 (0–0)	0.7 (0.3–1)	0 (0–0)	−7.62 (−8.57 to −6.65)
North Africa and Middle East	1524.4 (517.8–3596.5)	0.3 (0.1–0.7)	288.2 (95.3–1051.9)	0 (0–0.2)	−4.67 (−5.06 to −4.27)
Oceania	37.3 (6.4–101.6)	0.4 (0.1–1)	35.1 (6.4–90.6)	0.2 (0–0.4)	−1.94 (−2.09 to −1.78)
South Asia	2080.2 (252.2–6408.8)	0.1 (0–0.4)	252 (41.7–862.6)	0 (0–0.1)	−6.18 (−6.35 to −6.01)
Southeast Asia	975.2 (444.6–1913.4)	0.2 (0.1–0.3)	191.1 (82.8–363.2)	0 (0–0.1)	−4.98 (−5.05 to −4.91)
Southern Latin America	4 (1.9–8.5)	0 (0–0)	0.8 (0.3–1.4)	0 (0–0)	−5.09 (−5.99 to −4.18)
Southern Sub-Saharan Africa	62.2 (29.5–99.3)	0.1 (0–0.1)	36.2 (14.9–65.7)	0 (0–0.1)	−1.06 (−1.41 to −0.71)
Tropical Latin America	161.1 (112.8–198.6)	0.1 (0.1–0.1)	17.9 (11.1–25.3)	0 (0–0)	−6.17 (−6.65 to −5.68)
Western Europe	27.2 (19.9–33.1)	0 (0–0)	1.3 (0.6–2)	0 (0–0)	−8.76 (−9.84 to −7.68)
Western Sub-Saharan Africa	350 (25.1–1981.4)	0.1 (0–0.5)	402.8 (45.7–1900.4)	0 (0–0.2)	−1.32 (−1.56 to −1.07)
DALYs					
Global	1290532.6 (590788.8–2246406.8)	20.8 (9.7–35.8)	408775.3 (252320.1–671119.9)	5.8 (3.5–9.8)	−4.13 (−4.28 to −3.98)
High SDI	30744.8 (18908.9–44317.2)	4.3 (2.5–6.3)	22030.9 (13635.6–33496.9)	2.2 (1.4–3.4)	−2.09 (−2.31 to −1.87)
High-middle SDI	279207.1 (124269.2–527212.6)	31.1 (13.6–59.3)	32495.7 (21979.2–48008.9)	3.2 (2.2–4.5)	−8.17 (−8.57 to −7.76)
Middle SDI	519928.6 (260467.9–850007.8)	26.4 (13.4–42.9)	92289.2 (65013.8–129542.5)	4.4 (3.1–6.1)	−6 (−6.22 to −5.79)
Low-middle SDI	325981.7 (132397.4–734145.2)	19.5 (8.7–42.2)	134879.5 (88416.2–201658.2)	7.1 (4.6–10.6)	−3.19 (−3.25 to −3.14)
Low SDI	134026.9 (30708.7–446529.9)	15.2 (4.7–46.7)	126685.1 (49746.3–344500.6)	8.6 (3.9–21.7)	−1.47 (−1.64 to −1.31)
Andean Latin America	17436.4 (9006.3–28478.7)	32.3 (17.1–52)	3210.6 (2335.8–4508.4)	5.1 (3.7–7.1)	−6.01 (−6.44 to −5.59)
Australasia	421 (270.9–627.5)	2.2 (1.4–3.3)	550.9 (348.8–839.2)	1.9 (1.2–2.9)	−0.36 (−0.45 to −0.26)
Caribbean	2,540 (1079.4–5389.4)	6.2 (2.8–12.9)	2049.2 (1218–3,329)	4.7 (2.7–8)	−0.66 (−0.75 to −0.56)
Central Asia	6537.1 (4575.6–9347.1)	8.2 (5.8–11.8)	5597.1 (3769.2–8351.8)	5.8 (3.9–8.6)	−1.34 (−1.51 to −1.17)
Central Europe	4336.3 (3101.5–5967.2)	4.2 (3–5.8)	1941 (1198.8–2954.3)	1.8 (1.2–2.8)	−2.66 (−2.82 to −2.5)
Central Latin America	30895.5 (19981.1–38687.1)	13.7 (9–17.1)	9694.7 (6999.8–13400.7)	4.4 (3.2–5.9)	−3.8 (−3.98 to −3.63)
Central Sub-Saharan Africa	8727.1 (2202.8–33257.7)	8.5 (2.8–29.6)	8642.3 (3613.9–24278.8)	4.8 (2.2–12.1)	−1.52 (−1.88 to −1.17)
East Asia	567452.8 (210661.3–1071789.9)	49.3 (18.2–93.3)	36,372 (24862.6–52967.5)	3.6 (2.4–5.2)	−9.41 (−9.93 to −8.88)
Eastern Europe	7,111 (4966.3–9801.3)	3.8 (2.6–5.3)	3537.9 (2200.8–5391.7)	1.9 (1.3–2.9)	−2.64 (−2.83 to −2.45)
Eastern Sub-Saharan Africa	47522.3 (10182.3–198395.6)	13.2 (3.8–50.4)	36,757 (14299.3–113,593)	6.6 (2.9–18.4)	−1.94 (−2.07 to −1.81)
High-income Asia Pacific	7122.9 (4645.9–10,330)	5 (3.2–7.6)	5517.1 (3380.1–8453.6)	3.3 (2–5.1)	−1.22 (−1.41 to −1.02)
High-income North America	4601.7 (3223.7–6497.1)	1.8 (1.3–2.5)	4438.1 (2676.2–6902.9)	1.3 (0.8–2)	−1.53 (−1.89 to −1.16)
North Africa and Middle East	156136.9 (66577.3–340261.1)	31.8 (14.5–67.4)	58524.5 (34890.2–125071.9)	9.7 (5.7–21.2)	−3.13 (−3.41 to −2.85)
Oceania	3455.6 (674.2–9259.6)	33.1 (6.9–87.9)	3446.5 (859–8439.7)	17.5 (4.8–42.2)	−1.73 (−1.87 to −1.59)
South Asia	256415.1 (82364.5–641525.1)	17.6 (6.6–41.4)	122621.6 (76631.1–193338.1)	6.9 (4.2–11)	−2.99 (−3.07 to −2.92)
Southeast Asia	96169.7 (48892.7–181342.7)	16.7 (8.7–31.3)	35296.7 (23249.2–53115.9)	5.8 (3.7–9)	−3.49 (−3.61 to −3.37)
Southern Latin America	1225.7 (822.3–1797.4)	2.4 (1.6–3.6)	1114.1 (711.8–1711.8)	1.8 (1.2–2.7)	−1.07 (−1.16 to −0.98)
Southern Sub-Saharan Africa	7652.6 (4591.1–10780.2)	11 (6.9–15.2)	6,098 (3886.3–8970.3)	7.7 (4.9–11.4)	−0.7 (−0.92 to −0.48)
Tropical Latin America	17356.4 (12612.5–21039.9)	10.8 (7.9–13.1)	4984.9 (3585.1–7008.7)	2.5 (1.8–3.4)	−4.22 (−4.65 to −3.78)
Western Europe	9400.7 (6764.6–13053.4)	3 (2.2–4)	6841.7 (4201.1–10342.1)	1.7 (1.1–2.6)	−1.57 (−1.82 to −1.32)
Western Sub-Saharan Africa	38015.8 (8145.1–184706.9)	10.9 (3.2–47.2)	51539.5 (18210.1–184528.5)	7.3 (3–23.3)	−0.92 (−1.09 to −0.75)

### Regional level

From the perspective of social development level, OFCs burden worldwide shows notable regional differences, and the disease burden indicators vary greatly among different SDI regions. The ASPR in low-middle SDI regions represented to be the highest, of 76.3 per 100,000 (95% UI: 60.7–93.6), and regions with high SDI reported the lowest, of 33.6 per 100,000 (95% UI: 27–40.6). The EAPC of the two regions is similar, with the former being −0.23 (95% CI: −0.29 to −0.16) and the latter being −0.23 (95% CI: −0.25 to −0.22). ASPR dropped in all regions, with the middle-high SDI region showing the most notable drop, showing EAPC of −0.46 (95% CI: −0.52 to −0.39). ASPR has decreased from 40.4 per 100,000 (95% UI: 32.9–48.3) in 1990 to 36.2 per 100,000 (95% UI: 29.6–43.5) in 2021, indicating more significant progress in the prevention and treatment of OFCs in these regions compared to other areas. The EAPC of the low SDI region is −0.29 (95% CI: −0.32 to −0.26), and its ASPR has decreased from 68.9 per 100,000 (95% UI: 55–83.8) in 1990 to 62.8 per 100,000 (95% UI: 50.5–76.8). The EAPC within middle-SDI region is −0.07 (95% CI: −0.1 to 0.04), which is almost the same as the ASPR in 1990 and 2021 (Table 1; Figure 1).

ASIR also shows regional differences. High SDI regions exhibited the lowest ASIR at 2.5 per 100,000 (95% UI: 1.8–3.2), and middle-low SDI regions showed the highest at 3.3 per 100,000 (95% UI: 2.5–4.4). The analysis of the ASIR reveals notable trends. The EAPC in the high-middle SDI regions is the highest, reaching −1.53 (95% CI: −1.59 to −1.47), while other regions exhibit a more uniform EAPC of around −0.85 (Table 1; Figure 1). The ASDR in low SDI areas is the highest, reaching 0.1 per 100,000 (95% UI: 0–0.2), whereas other regions are nearly at zero. Similarly, the EAPC for high-middle SDI areas is the highest, at −11.62 (95% CI: −12.12 to −11.11) (Table 1; Figure 1). The age-standardized DALYs rate further revealed regional differences. Low SDI regions have the highest DALYs rate at 8.6 per 100,000 (95% UI: 3.9–21.7), while high SDI regions have the lowest at 2.2 per 100,000 (95% UI: 1.4–3.4). Notably, high-middle SDI regions show the most significant decrease in age-standardized DALYs rate, with an EAPC of −8.17 (95% CI: −8.57 to −7.76) (Table 1; Figure 1).

Global data demonstrate a substantial 31-year decline (1990–2021) in OFCs disease burden, primarily attributable to advancements in medical technology and socioeconomic development. High-middle SDI regions exhibited particularly notable progress, reflecting accelerated improvement in therapeutic and preventive capability once society reaches this developmental threshold. Indicators of disease burden in high-SDI regions maintained the lowest level over three decades, due to their well-established superior healthcare infrastructure and social conditions since 1990. Conversely, the paradoxically lower prevalence and incidence rates observed in low-SDI regions are likely attributable to severe underreporting primarily driven by neonatal mortality among untreated cases and systemic gaps in health surveillance systems (20). Despite incremental advancements in low- and low-middle SDI regions, OFCs continues to impose disproportionately heavy health burden in these areas.

**Figure 2:**
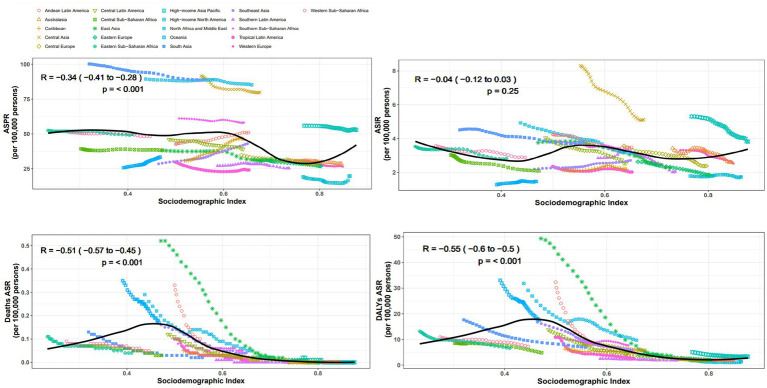
Association between SDI and disease burden indicators for OFCs in 21 regions.

From the perspective of geography, the top three regions with the highest ASPR in 2021 are South Asia, North Africa and Middle East, Central Asia, the values are, respectively, 89.1 per 100,000 (95% UI: 70.7–110.1), 85.2 per 100,000 (95% UI: 68.2–103.5), and 80 per 100,000 (95% UI: 64.6–96.4). In contrast, high-income North America had the lowest ASPR at 19.7 per 100,000 (95% UI: 15.1–24.9). ASPR in the Australasia and Europe are significantly lower than those in Asia and Africa. Almost all regions experience a decline in prevalence between 1990 and 2021, except for the four regions: Andean Latin America, East Asia, Caribbean, Southeast Asia, with EAPCs of 1.63 (95% CI: 1.54 to 1.72), 1.32 (95% CI: 1.24 to 1.4), 0.82 (95% CI: 0.78 to 0.86), and 0.6 (95% CI: 0.54 to 0.65), respectively (Table 1). Additionally, we conducted a correlation analysis between ASPR and SDI across varying areas, incorporating changes in SDI from 1990 to 2021. The results are shown in Figure 2. We used distinct shapes and colors to represent different regions and plotted 21 lines based on their SDI changes over this period. The computation outputted negative relation between ASPR and SDI, with a correlation coefficient of −0.34 (95% CI: −0.41 to −0.28) and *p*-value < 0.001 (Figure 2).

Despite the most significant decline in ASIR in Central Asia over the past 32 years, with an EAPC of −1.75 (95% CI: −1.83 to −1.67), its ASIR still ranks highest at 5.1 per 100,000 (95% UI: 3.6–6.9). South Asia and high-income Asia Pacific follows closely, with rates of 3.8 per 100,000 (95% UI: 2.7–5.1) and 3.8 per 100,000 (95% UI: 2.5–5.2), respectively. The region with the lowest ASIR is Oceania, at 1.5 per 100,000 (95% UI: 1.0–2.1). The regions with positive EAPC values include Southeast Asia Oceania, Caribbean, high-income North America, with values of 0.67 (95% CI: 0.62 to 0.73), 0.38 (95% CI: 0.3 to 0.46), 0.28 (95% CI: 0.16 to 0.41), and 0.01 (95% CI: −0.09 to 0.12), respectively (Table 1). The correlation coefficient between SDI values and ASIR across different regions is −0.04 (95% CI: −0.12 to 0.03), with a *p*-value of 0.25, indicating no significant correlation (Figure 2).

Oceania has the highest ASDR at 0.2 per 100,000 (95% UI: 0–0.4), while in other regions the value is around zero. During the 32 years, ASDR has significantly decreased in all regions (Table 1). The correlation coefficient between SDI values and ASDR is −0.51 (95% CI: −0.57 to −0.45), with a *p*-value of<0.001, indicating a significant correlation (Figure 2).

The top three rates of age-standardized DALYs in 2021 are Oceania, North Africa and Middle East, Southern Sub-Saharan Africa, at 17.5 per 100,000 (95% UI: 4.8–42.2), 9.7 per 100,000 (95% UI: 5.7–21.2), and 7.7 per 100,000 (95% UI: 4.9–11.4), respectively. High-income North America has the lowest rate at 1.3 per 100,000 (95% UI: 0.8–2.0). Rates of age-standardized DALYs have dropped within all regions, with East Asia showing the most notable decline, at an EAPC of −9.41 (95% CI: −9.93 to −8.88) (Table 1). The SDI value of each region shows negative relation with rate of age-standardized DALYs, with a correlation coefficient of −0.55 (95% CI: −0.60 to −0.50), *p*-value < 0.001 (Figure 2).

### National level

**Figure 3:**
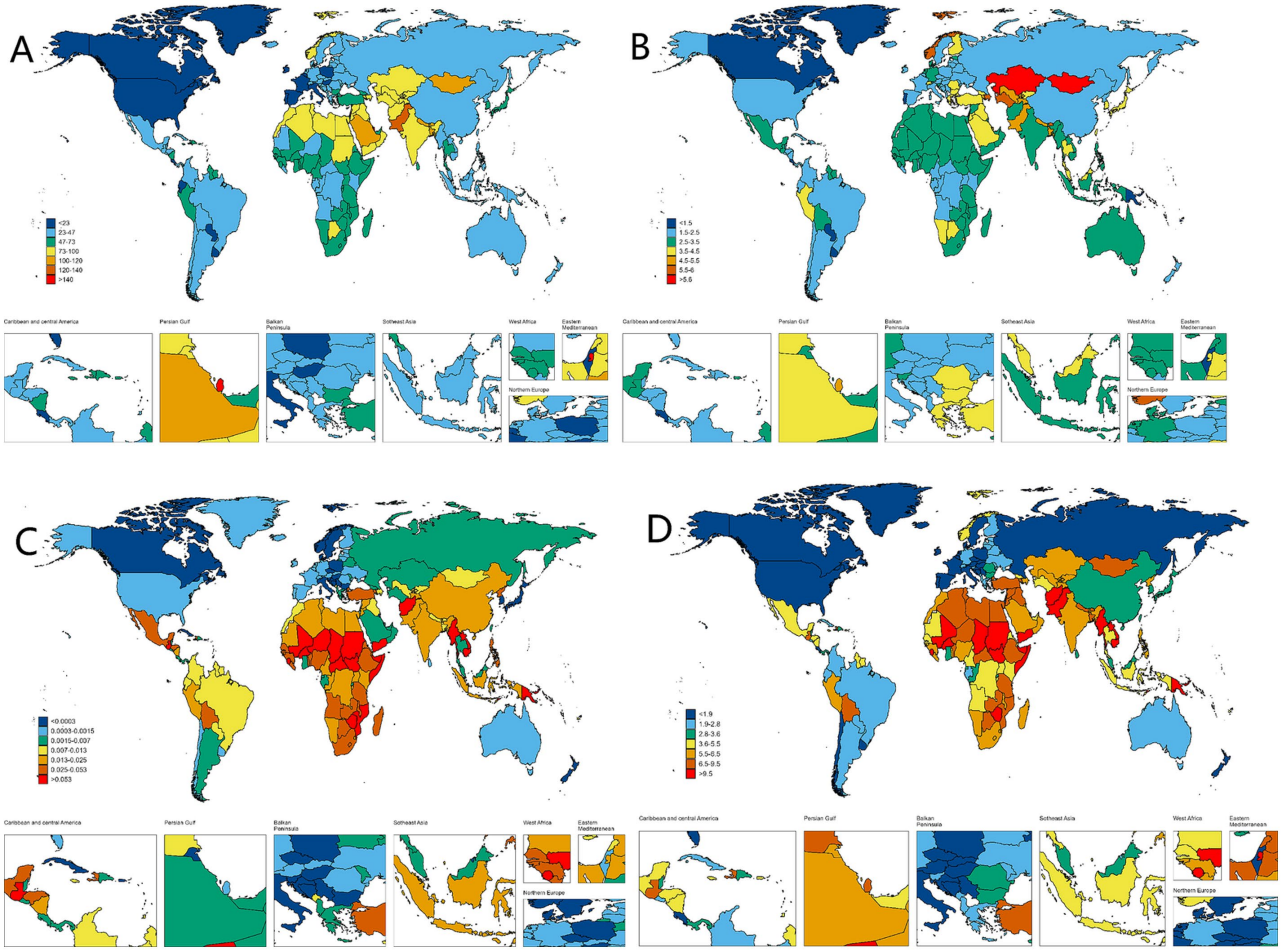
Worldwide health impact of OFCs among both genders across 204 nations and regions. **(A)** Prevalence rate. **(B)** Incidence rate. **(C)** Death rate. **(D)** DALYs rate.

**Figure 4:**
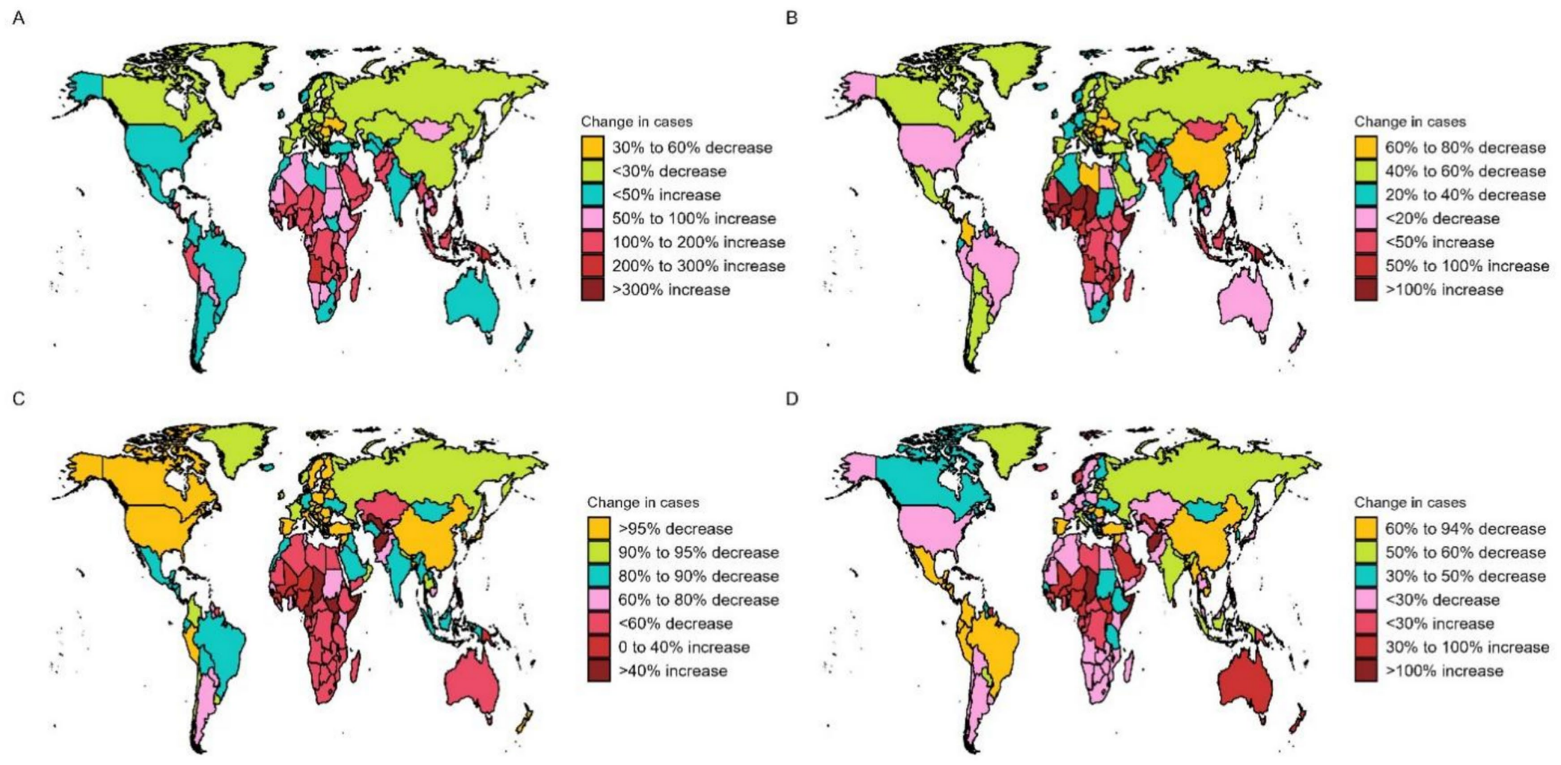
OFCs cases for both genders across 204 nations and regions. **(A)** Change prevalence cases. **(B)** Change incidence cases. **(C)** Change deaths cases. **(D)** Change DALYs.

**Figure 5:**
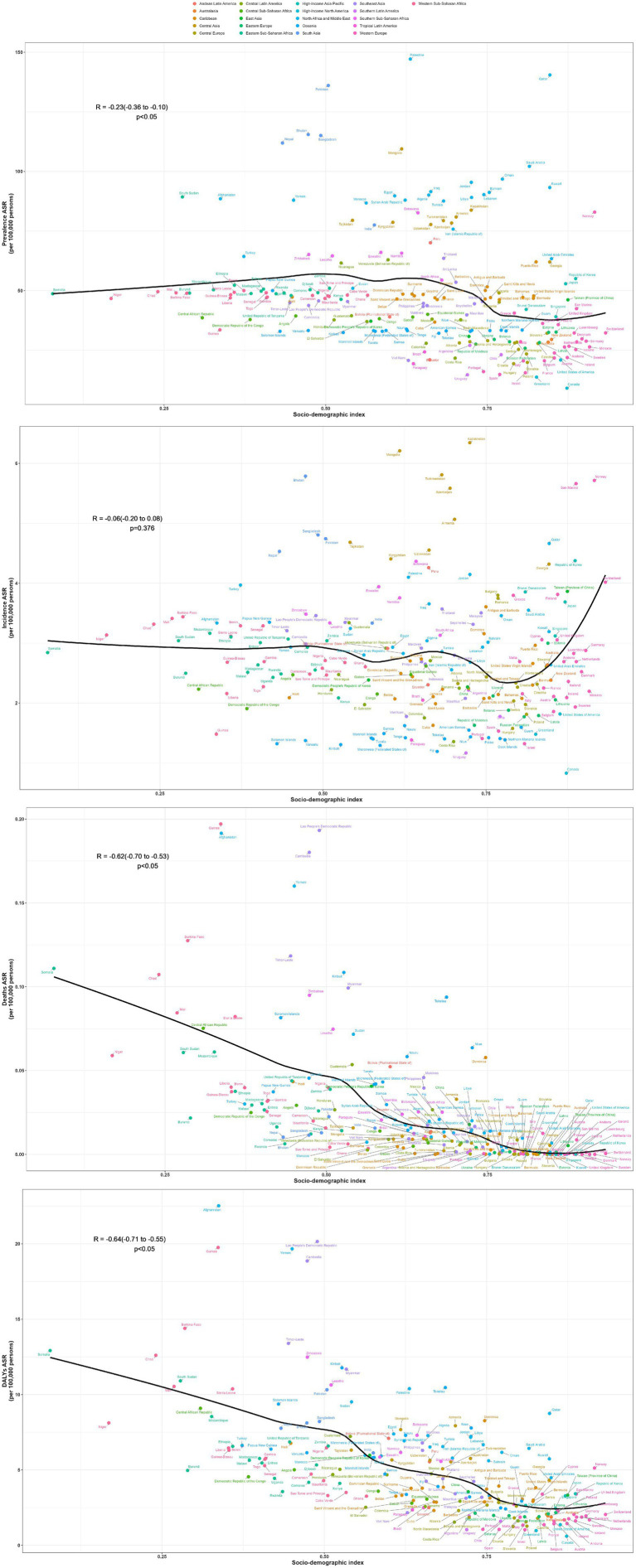
Association between SDI and disease burden indicators for OFCs in 204 countries.

In 2021, countries with the top three high ASPR were Palestine (147.15 per 100,000, 95% UI: 119.61–176.54), State of Qatar (140.47 per 100,000, 95% UI: 111.24–173.15), and Islamic Republic of Pakistan (136.06 per 100,000, 95% UI: 107.62–169.11), all of which are in Asia. The country with the lowest ASPR is Canada (9.07 per 100,000, 95% UI: 7.15–11.05). Puerto Rico is the country with the most notable elevation in ASPR (EAPC: 2.05, 95% CI: 1.93 to 2.18), while Italy has the most notable decline in ASPR (EAPC: −1.36, 95% CI: −1.55 to −1.17) (Supplementary Table S1; Figure 3A). The fastest elevation in case number is in Qatar, with a 534.2% elevation in case number in 2021, compared to 1990 (Figure 4A). We conducted several correlation analyses between these disease burden indicators and SDI values across different countries. Correlation coefficient between SDI and ASPR is −0.23, showing a negative correlation, with a 95% CI of −0.36 to −0.10 and a *p*-value < 0.05 (Figure 5).

In terms of ASIR, the top-ranked countries are Kazakhstan (6.34 per 100,000, 95% UI: 4.33–8.64), Mongolia (6.21 per 100,000, 95% UI: 4.00–9.25), and Turkey (5.81 per 100,000, 95% UI: 4.09–7.76), all situated in Central Asia. Conversely, Canada has the lowest ASIR at 0.83 per 100,000 (95% UI: 0.58–1.13). The ASIR of Taiwan (Province of China) has seen the most significant increase (EAPC: 2.95% CI: 1.73 to 2.28) (Supplementary Table S2; Figure 3B). Countries experiencing a rapid surge in the number of cases include Qatar, Papua New Guinea, Chad, Niger, Mali, and Somalia, with their incidence rates in 2021 more than doubling compared to 1990 (Figure 4B). The correlation coefficient between SDI values and ASIR is −0.06 (95% CI: −0.20 to 0.08), with a *p*-value of 0.376, indicating no clear correlation between the two (Figure 5).

Papua New Guinea has the highest ASDR (0.19 per 100,000, 95% UI: 0.04–0.52) (Supplementary Table S3; Figure 3C). Except for a few regions and countries with an increase in ASDR globally (e.g., Afghanistan, Trinidad, and Tobago), there has been significant decreases in ASDR in all others. It can be observed that countries with increased deaths, such as Afghanistan, Chad, Somalia, etc., are mainly located in Sub Saharan Africa and Central Asia (Figure 4C). The correlation coefficient between the SDI values and ASDR is −0.62 (95% CI: −0.70 to −0.53), with *p*-value <0.05, indicating a significant correlation (Figure 5).

The age-standardized DALYs rate is highest in Afghanistan (22.53 per 100,000, 95% UI: 5.8–104). The fastest decline is in China, with an EAPC of −9.59 and a 95% CI: −10.13 to −9.03 (Supplementary Table S4; Figure 3D). Likewise, the nations experiencing the most substantial increases are predominantly in Sub Saharan Africa and Central Asia (Figure 4D). The correlation coefficient between the SDI values and ASDR is −0.64 (95% CI: −0.71 to −0.55), *p*-value <0.05(Figure 5).

### Gender differences in disease burden

**Figure 6:**
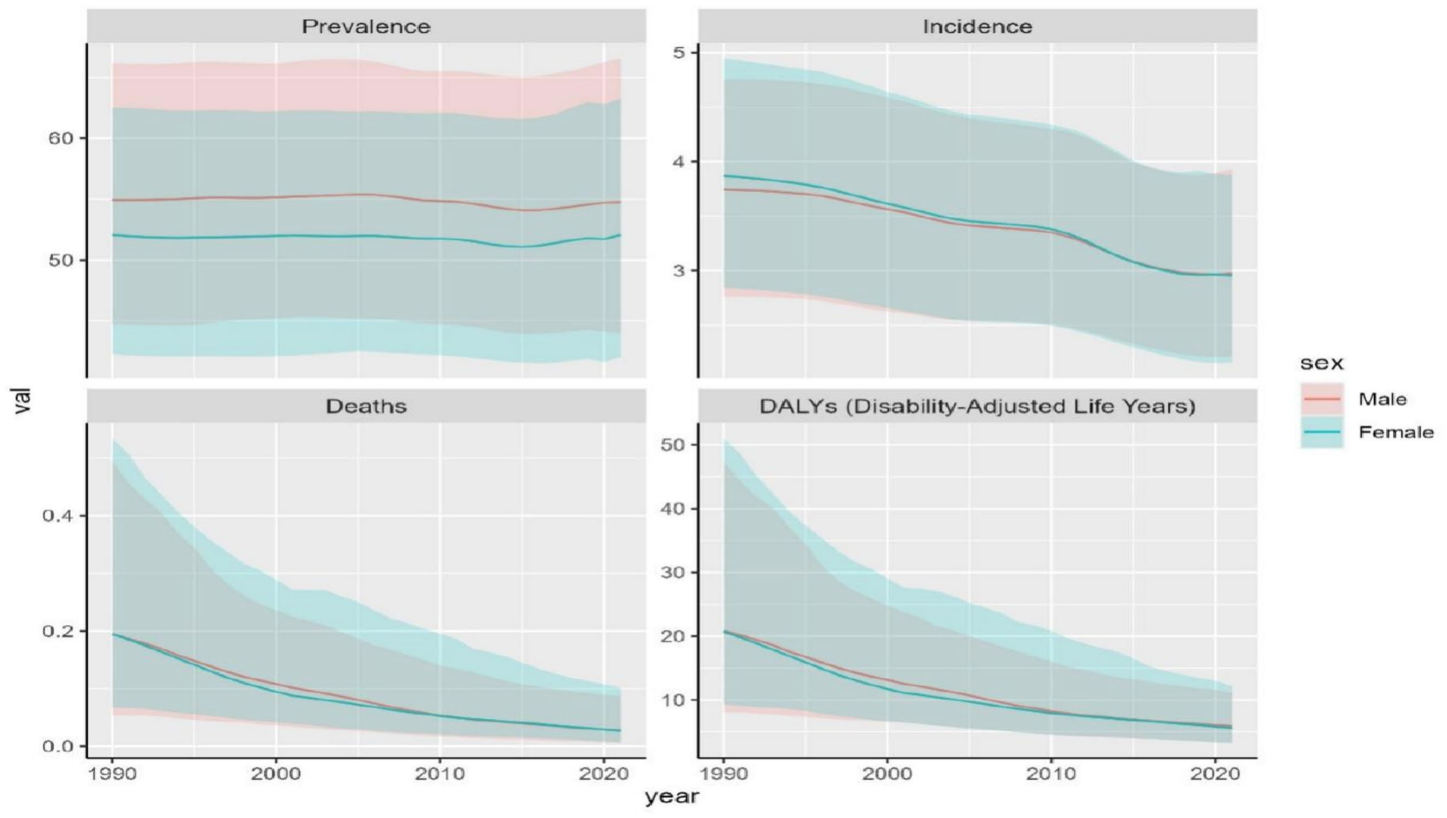
disease burden indicators for OFCs in males and females from 1990 to 2021.

Between 1990 and 2021, trends of OFCs disease burden showed nearly identical patterns between two genders. Male population exhibited higher ASDR, age-standardized DALYs rate, ASPR compared to females, while females exhibited higher ASIR. The disparity in ASPR between genders remained stable, with males maintaining higher ASPR values by 2021. In contrast, ASDR, age-standardized DALYs rate, and ASIR reached comparable levels between males and females by 2021 (Figure 6). Among the four disease burden indicators, only ASPR showed statistically significant gender differences (*p* < 0.001).

### Projections of the global impact of OFCs

**Figure 7:**
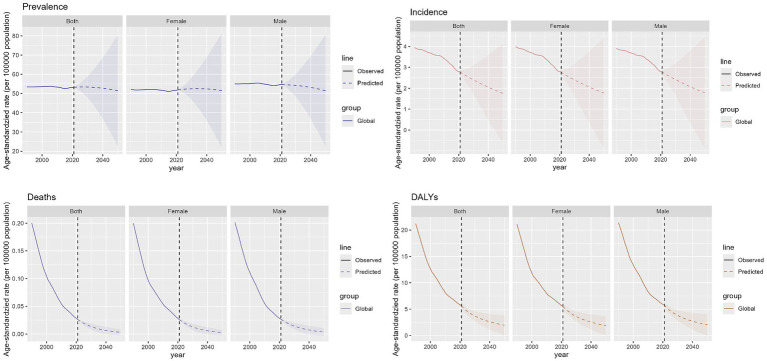
Future forecasts of global burden of OFCs.

We predict the trajectory of OFCs burden from 2022 to 2050. The combined ASPR for both sexes is anticipated to decline globally, falling from roughly 53.4 per 100,000 in 2021 to about 51.4 per 100,000 by 2050, which equates to a nearly 3.7% reduction over 30 years. Expected prevalence in male population is fairly constant, with minor rise from around 54.7 per 100,000 to 51.5 per 100,000, from 2021 to 2050. For females, a comparable decline is anticipated, with the prevalence rate falling from around 52.0 per 100,000 in 2021 to 51.5 per 100,000 by 2050. The global incidence of OFCs is also expected to experience a slight downward trend for both genders combined. ASIR is projected to decrease from approximately 2.81 per 100,000 in 2021 to about 1.78 per 100,000 by 2050. Additionally, ASDR for OFCs is expected to decline for both sexes combined, from roughly 0.026 per 100,000 in 2021 to around 0.003 per 100,000 by 2050. Moreover, rate of age-standardized DALYs for OFCs is expected to see a significant decline for the sum of both gender groups, falling from 5.8 per 100,000 to approximately 2.0 per 100,000, 2021 to 2050. In all, the decrease in the ASPR is relatively modest, and the data differences between males and females are minimal (Figure 7).

## Discussion

We investigated the trend of the disease burden of OFCs over time, as well as its relationship with factors (e.g., social development level and gender). Globally, notable reductions were seen in age-standardized DALYs rate, ASDR, ASIR, demonstrating enhanced capabilities in both preventive strategies and clinical management of OFCs. However, the disease burden of OFCs exhibits strikingly inequitable distribution and is inversely associated with the socio-demographic index. Regions with high SDI consistently demonstrate superior healthcare capabilities. Over the past three decades, three key indicators (ASPR, ASDR, and age-standardized DALYs rate) have remained stable and consistently lower than other regions, while only ASIR shows variability. This suggests that high-SDI regions not only deliver comprehensive, high-quality therapeutic interventions but have also achieved measurable advancements in primary prevention strategies for OFCs. In regions with high-middle SDI, rapid economic growth has been accompanied by the most significant reduction in disease burden. Notably, ASPR and ASIR in low-SDI areas are lower than those in low-middle SDI regions, potentially attributable to incomplete data-reporting system, inconsistencies in case definitions, or model-based misinterpretations of epidemiological patterns in resource-limited settings. Furthermore, correlation analyses across 21 geographical regions revealed that SDI values exhibited negative associations with ASPR, ASDR, and age-standardized DALYs rate, while no significant correlation was observed with ASIR.

Drawing on comprehensive analysis of the GBD 2021 data, our calculations indicate a global prevalence of OFCs in approximately 1 per 731 live births, statistically aligning with existing literatures (21). Central Asia stands out with a notably high incidence rate. Environmental pressures such as intense solar irradiance and significant diurnal temperature differences between day and night in Central Asia, may trigger abnormal gene expression during embryonic development (22, 23). Moreover, the traditional diet mainly consists of meat and dairy products, may lead to insufficient intake of vegetables and a consequent lack of key nutrients like folate among pregnant women (24). Additionally, exposure to secondhand smoke and the drinking habits of pregnant women are also known to contribute to the occurrence of OFCs (25). However, the age-standardized DALYs rate in Central Asian countries remain relatively low, which can attributed to the humanitarian activities of voluntary surgeons (26).

South Asia is characterized by high ASPR. The region has a large population base, and medical expenditure generally constitutes a low proportion of GDP (27). Meanwhile, some countries have received support from the WHO and have implemented measures to enhance prenatal care and early screening, leading to more cases being documented (28). The medical resources in this region are extremely scarce, with a severe shortage of surgeons, dentists, and nurses (29). This makes it challenging for patients to receive satisfactory treatment. Even when patients do receive treatment, their psychosocial trauma remains significant (30).

it’s the overall disease burden of Southeast Asia is lower than that of Central and South Asia, there are significant disparities among individual countries within the region. Vietnamese women generally consume less tobacco and alcohol, and their nutritional intake and infection prevention during pregnancy are relatively well-maintained, ensuring that children can receive timely and effective treatment (31). On the contrary, Laos faces a much heavier disease burden, with the second highest rate of age standardized DALYs worldwide in 2021, almost ten times that of Vietnam, with ASDR also ranking second in the world. In Laos, Indonesia, and the Philippines, the majority of OFCs patients are unable to receive timely and effective treatment (32–35).

East Asia has the lowest burden of OFCs disease in Asia. As the world’s second most populous country, China has witnessed rapid development, implemented policies related to eugenics and child rearing, and widely promoted prenatal checkups (36). Among high SDI region, high-income Asia Pacific exhibits the heaviest disease burden. Despite having extremely low birth rates in recent years, Japan and South Korea still exhibit relatively high ASIR of OFCs, second only to Central Asia. The elevated incidence may be linked to advanced maternal age and specific dietary patterns. Some scholars have found that excessive intake of multiple vitamins in the early stage of pregnancy can also lead to the occurrence of OFCs (37). Moreover, the high aesthetic standards in Korean society may impose greater psychological stress on individuals with OFCs in their daily lives (38).

OFCs disease burden remains relatively high in Middle East and North Africa. The traditional diet in these areas mainly consists of meat and cereals, women of childbearing age and pregnant women significantly lack folate intake (39). High proportion in women population used hookah during pregnancy, and the number of people with gestational diabetes is larges (40). Some communities still practice consanguineous marriages (41). Moreover, the unstable political situation is also one of the major challenges, such as Afghanistan, whose age-standardized DALYs rate ranks first among all countries, indicating that patients are unable to receive ideal treatment (42).

Sub Saharan Africa experiences rapid population growth, with approximately 1/778 of newborns affected by OFCs. This is significantly different from the previously ratio of 1/2500 in African newborns (43). This may be due to the previous excessive scarcity of medical resources in Africa, coupled with cultural superstitions that led to the discrimination against OFCs patients (44, 45). As a result, many children did not have the opportunity to seek medical treatment and were not recorded (46). Africa’s medical resources remain extremely limited, with low medical insurance coverage and a heavy reliance on international funding and policy support. Many children with OFCs miss out on surgical opportunities due to insufficient nutritional intake and feeding difficulties caused by cleft lip and palate. Their weight often fails to meet the requirements for surgery, causing them to miss the optimal treatment window (47–49).

In Oceania, the impact caused by OFCs is significant, which is primarily due to the overwhelming disease burden in Papua New Guinea. The country has a notably high rate of teenage pregnancy (50). Meanwhile, tropical climate and poor hygiene conditions exacerbate the risk of infections during pregnancy (51). Papua New Guinea is also home to numerous volcanoes, and the volcanic ash emissions contain cadmium, a heavy metal linked to a higher risk of OFCs (52). Medical resources in the country are extremely scarce and unevenly distributed. Although international medical aid teams provide free surgeries, coverage remains low due to inadequate transportation and postoperative rehabilitation resources (53).

Thanks to sufficient medical resources and effective perinatal management (54), the disease burden in Australasia and Europe remains quite low. High-income North America has the lowest disease burden. The disease burden in Latin America and the Caribbean region is roughly at a moderate level, OFCs distribution in Latin American and Caribbean countries presented to be heterogeneous, with no geographic pattern (55). Brazil has a relatively high rate of tobacco and alcohol exposure during pregnancy, a higher risk of infections, and a high prevalence of hypertension, along with lower coverage rate of surgical procedures (56, 57). In addition, some studies suggest that the abuse of cannabis and other drugs in Latin America is also associated with OFCs occurrence (58).

From a global perspective, the disease burden of men is slightly higher than that of women. This phenomenon may be the result of the combined effects of genetics and embryonic development (59). From a genetic perspective, some genes exhibit stronger dominant effects in male embryos (60). In terms of embryonic development, the critical period of facial fusion in male embryos is more sensitive to external interference (19). In addition, male patients are more likely to be detected in statistics due to a higher proportion of bilateral complete cleft lip and palate, while female embryos have lower tolerance for severe deformities, which may lead to a higher rate of early natural elimination (61).

We predict that the disease burden of OFCs will continue to decrease in the future, and in order to accelerate this process, tailored interventions should be implemented based on country-specific disease burden profile. In areas with relatively low SDI, the disease burden is significantly heavier. For congenital disorders such as OFCs, prevention and early treatment are crucial in these areas. It requires implementing prenatal and eugenic policies to prohibit consanguineous marriages, enhancing prenatal screenings during pregnancy, ensuring adequate nutrition supply, preventing infections, getting rid of superstition, and avoiding medication misuse (62–65). For the care of newborns with OFCs, it is essential to ensure prompt feeding care, prevent lower respiratory tract infections, manage psychomotor retardation, and treat of comorbidities (66–69).

Strengthening horizontal health systems is likely the optimal long-term strategy compared to vertical, disease-specific interventions for addressing cleft-related disabilities (20). Ensuring timely and effective access to basic surgical care for patients is the most cost-effective approach (43, 70, 71). It not only reduces the need for subsequent corrective treatments but also effectively alleviates the disease burden and prevents the waste of medical resources (72). For instance, prenatal intrauterine surgical repair can achieve nearly scar-free healing (73). Meanwhile, it is also crucial to establish effective disease surveillance and reporting systems in these regions to provide reliable epidemiological data.

Developed regions with high SDI have made sufficient efforts in many aspects, and their disease burden of OFCs is relatively light. Their main problem is delayed childbearing age, as parental age may be related to the occurrence and severity of OFCs (74). These regions should take the lead in establishing a global epidemiological surveillance system and initiating international support projects to assist underdeveloped areas (26), simultaneously conducting more high-quality multicenter studies to clarify the epidemiological characteristics and etiology of OFCs.

### Limitation

The GBD data rely on model estimates, which may introduce information bias due to variations in disease definitions, diagnostic criteria, and data collection methods (e.g., inconsistent reporting standards across national health departments). These factors can lead to overestimation or underestimation of disease burdens in specific regions, particularly in countries/regions with underdeveloped healthcare systems or weak data reporting mechanisms. Additionally, the database lacks detailed granular data (e.g., race/ethnicity, disease subtypes), limiting in-depth analyses for specific populations.

## Conclusion

Globally, the disease burden of OFCs has shown a decline from 1990 to 2021. Males have experienced a slightly higher disease burden compared to females, although the gap remains relatively narrow. The distribution exhibits significant regional disparities correlated with SDI values. The heaviest disease burden occurs in Central Asia, South Asia, and Africa. In these regions, it is crucial to strengthen three-tier prevention (preconception screening, prenatal diagnosis), enhancing primary healthcare sequential treatment capacity, integrating public welfare programs to offer free surgeries, collaborating with multidisciplinary teams for full-cycle rehabilitation, and promoting preventive knowledge through community education. Developed regions with lighter disease burdens should conduct research on cleft lip and palate, establish efficient and reliable epidemiological surveillance systems, and provide support to other regions.

## Data Availability

Publicly available datasets were analyzed in this study. This data can be found here: GBD study 2021 data resources were available online, the website is as follows: https://vizhub.healthdata.org/gbd-results/.
